# Comparison of clinical and cost-effectiveness of two strategies using mobile digital x-ray to detect pulmonary tuberculosis in rural India

**DOI:** 10.1186/s12889-019-6421-1

**Published:** 2019-01-22

**Authors:** Bornali Datta, Ashish Kumar Prakash, David Ford, Praveen K. Tanwar, Pinky Goyal, Poulomi Chatterjee, Smita Vipin, Anand Jaiswal, Naresh Trehan, Kavita Ayyagiri

**Affiliations:** 1 Department of Respiratory and Sleep Medicine, Medanta the Medicity, Gurgaon, India; 2 Department of Respiratory Medicine, Scarborough District Hospital, Scarborough, UK; 3 Al Afia Hospital Mandikhera, Mewat, India; 4 International Union Against Tuberculosis and Lung Disease, The Union South East Asia Office, New Delhi, India

**Keywords:** Public-private partnership, Active case finding, Tuberculosis, digital x-ray

## Abstract

**Background:**

Medanta - The Medicity, a multi-super specialty corporate hospital in Gurugram, Haryana launched a “TB-Free Haryana” Campaign; mobile van equipped with a digital CXR machine to screen patients with presumptive Tuberculosis (TB). Objectives: In this study, we aimed to assess the (1) yield and cost analysis of two strategies using mobile digital x-ray to detect Pulmonary TB in rural Haryana.

**Methods:**

An observational study was conducted on all individuals screened by either of the two case finding strategies using a mobile x-ray unit (MXU) mounted on a mobile van in District Mewat, Haryana during Jan-March 2016.

**Results:**

Strategy 1: Out of 121 smear negative cases, x-rays were suggestive of TB in 39(32%), of which 24 were started on TB treatment. Cost of identifying a smear negative TB was US$ 32. Strategy 2: Out of 596 presumptive TB, chest x-rays were suggestive of TB in 108 (18%), of which 67 were started on TB treatment (56 were smear negative TB). Cost of detecting any case of TB was US$ 08 (1 USD = 64 INR).

**Conclusion:**

The study reports a new initiative within a PPM model to improve the diagnosis of PTB by filling the gap in the current diagnostic infrastructure. We believe there is potential for replication of strategy 2 model in other states, although further evidence is required.

**Electronic supplementary material:**

The online version of this article (10.1186/s12889-019-6421-1) contains supplementary material, which is available to authorized users.

## Background

A total of 10.0 million cases of Tuberculosis (TB) were estimated in 2017, of which only 6.4 million (64%) were diagnosed and notified to national programs [1]. In order to achieve the World Health Organization (WHO) goal of ending the TB epidemic by 2035, we need to identify these missing TB cases and provide appropriate care to them. One of the strategies to identify these missing cases is early diagnosis and systematic screening of contacts and high-risk groups, also known as active case finding (ACF). ACF is defined as the “systematic identification and screening of people with presumptive TB, using tests, examinations or other procedures that can be applied rapidly” [2]. Some of the ACF interventions include screening of household contacts of index TB patients, screening of other high risk groups such as people living with HIV, prisoners, slum dwellers etc. and mass community screening of asymptomatic individuals.

India is one of the 20 high TB burden countries with estimated 2.47 million new TB cases and 421,000 deaths in 2017 [1]. Of the estimated new TB cases, only 65% were notified to the national TB program (NTP) in 2017 [1]. To reduce this gap, the National Strategic Plan 2017–2025 has recommended community-based ACF which includes community mobilization, symptom screening and referral [3].

Few randomized trials have assesses the impact of these ACF interventions on TB burden with conflicting findings. The DETECTB study, a cluster randomized trial in Zimbabwe evaluated an intervention involving screening of 100,000 adults with 2 weeks of cough, using either mobile vans or door-to-door visits. The study demonstrated a decline in disease prevalence from 6.5 to 3.7 per 1000 adults in a before-after study design [4]. The Zambia South Africa Tuberculosis and AIDS Reduction (ZAMSTAR) community trial showed no effect of the enhanced case finding (ECF) intervention on the incidence of TB [5]. A large door-to-door ACF activity under Project Axshya covering ~ 20 million people across 300 districts in India resulted in the detection of a large number of persons with presumptive pulmonary TB and smear-positive TB [6]. Different ACF strategies should be evaluated for their effectiveness to establish the most efficient and effective intervention and inform policy.

Medanta - The Medicity, one of India’s largest multi-super specialty corporate hospital located in Gurugram, Haryana launched a “TB-Free Haryana” Campaign in collaboration with the state Government of Haryana, India. Under this public–private partnership (PPP), a mobile van was equipped with a digital CXR machine with the support from other private players. The van was sent to a government health facility every week. The van used to screen patients with presumptive TB using a “health camp” approach. Two strategies of case finding were used. In strategy 1, adult patients (18 years and above) who, according to the Revised National Tuberculosis Control Programme (RNTCP) diagnostic algorithm were eligible but not able to get a CXR (chest symptomatic with sputum smear negative) underwent CXR. In strategy 2, all chest symptomatic patients (cough > 2 weeks) were called for chest x-ray, regardless of their smear status. Patients with abnormal x-ray findings were recalled for sputum testing. This strategy also involved active information education communication (IEC) and community mobilization one week prior to the case finding activity.

In this study, we aimed to assess the (1) yield and cost analysis of two strategies using mobile digital x-ray to detect PTB in rural Haryana, and (2) impact of the case finding project on overall case notifications in district Mewat, Haryana.

## Methods

### Study design and population

An observational study was conducted on all individuals screened by either of the two case finding strategies using a mobile x-ray unit (MXU) in District Mewat, Haryana during January 2016 to March 2016.

### General setting

The study was carried out in District Mewat, one of the 22 districts of Haryana state in Northern India inhabited by nearly 1.08 million people. Majority (95%) of the population lives in rural areas. It has a sex ratio of 906 females for every 1000 males and a literacy rate of 56.1% [7]. The district has 33 public health facilities including 01 District Hospital, 03 Community Health Centres (CHCs), 06 Primary Health Centres (PHCs) and 23 Sub-centres (SCs) [8].

### Specific setting

Medanta the Medicity, a corporate hospital in Gurgaon in collaboration with the Government of Haryana launched the “TB Free Haryana” campaign. A key strategy was of this campaign was identification of missing TB cases in the rural remote communities and put them to appropriate care. A mobile van equipped with a digital CXR machine visited a government health facility every week. An x-ray technician, nurse and a driver accompanied the van. Two strategies of active case finding were employed using a “health camp approach”.

*Strategy 1*: Four peripheral health facilities were visited during Jan and Feb 2016. The medical officer of the health facility was informed in advance of the arrival of the mobile van. Adult patients (18 years and above) who, according to the Revised National Tuberculosis Control Programme (RNTCP) diagnostic algorithm were eligible but not able to get a CXR (chest symptomatic with sputum smear negative) were requested to assemble at the health facility on the designated date of visit of mobile unit. The health system staff ensured that the eligible patient made the visit. The chest x-ray interpretation was done by the District TB Officer who is a qualified chest physician or by the consultant of the Department of Respiratory Medicine at Medanta. Those with findings consistent with active TB (apical infiltrates, cavity, miliary nodules, pleural effusion—in corroboration with appropriate clinical findings) were diagnosed as smear negative pulmonary TB and were initiated on treatment as per RNTCP guidelines. The details of this strategy has been described elsewhere [9].

#### Strategy 2

The second strategy of enhanced case finding involved active information education communication (IEC) one week prior to the case finding activity. Pamphlets were distributed in the villages by the accredited social health activists (ASHAs). Banners were displayed in the health facility and radio/newspaper announcements were made to increase awareness amongst village residents both about the disease and about the visit of the mobile x-ray van. The RNTCP staff at district and Tuberculosis unit (TU) level, sarpanch and key local members was also involved in mobilizing the community. The mobile van visited 12 peripheral health institutes (PHIs) during Jan to March 2016. In this strategy, all chest symptomatic patients (cough > 2 weeks) underwent chest x-ray, regardless of their smear status. The reason for implementing Strategy 2 was the relatively low turnout of patients with Strategy 1. Strategy 2 followed new RNTCP guidelines 2016, which also recommend the use of chest x-ray where available, alongside sputum examination. A detailed proforma was filled for each patient recording all relevant details. Patients with abnormal x-ray findings were recalled for sputum smear microscopy. X-ray findings, sputum results, clinical presentation and previous TB status were taken into consideration by the doctor at the health facility before diagnosis of TB and starting treatment.

### Data variables and source of data

Aggregate data (number of persons who underwent screening i.e. x-ray and/or sputum examination, number of persons with positive/negative x-ray finding and positive/negative smear result, number of persons started on DOTS) and cost estimates were collected from project records. Other variables such as socio-demographic (age, sex) and clinical characteristics (presence of symptoms such as cough, fever, weight loss, hemoptysis) and x-ray finding were also extracted from project records.

### Cost analysis

Cost items were grouped into six categories: mobile van, x-ray equipment, personnel, operating costs (van insurance, annual maintenance costs and fuel), Information Education and Communication (for strategy 2 only) and miscellaneous. Cost estimates were obtained from project financial reports at 2016 prices. USD conversion rate of 2016 was used (1 USD = 64 INR).

### Van and equipment

The one-time cost of van including fabrication (Rs 800,000) was estimated to be Rs 2,300,000 and for the equipment at Rs 620,000. The equipment consists of an Allenger’s 30 mA portable x-ray machine and a Fuji digital reader for digital review and printing of the x-ray. The mobile van and x-ray equipment were considered capital purchases, and only the annual depreciation was incorporated into the analysis. A depreciation time of 10 years was used for the analysis for the van and 7 years for the equipment. Operating costs included annual maintenance of the equipment, van insurance and fuel. The annual insurance for the mobile van was estimated at Rs 25,000. The details of the cost of various components are given in Additional file 1: Appendix 1.

### Personnel

A nurse, a radiographic technologist (RT) and a driver were employed at Rs 15,000 per month.

### Cost-effectiveness analysis

Considering five working days a week and two additional off days a month, a total of 18 camps are expected each month. A total of 13 and 50 patients visited each camp under strategy 1&2 respectively during the study period (first quarter of 2016). For strategy 1, total cost was divided by 2808 (13 patients per camp*18 camps/month*12 months) and for strategy 2, total cost was divided by 10,800 (50 patients per camp*18 camps/month*12 months) to get the cost per screening. In strategy 2, only 67 had their sputum tested among 108 with x-ray suggestive of TB (after excluding those already on treatment). All of them were started on TB treatment (11 smear positive and 56 smear negative). Sensitivity analysis was also performed examining cost estimates at different efficiencies of the mobile van in terms of the number of patients visiting each camp.

### Analysis and statistics

Data were entered into Microsoft Excel and then imported into EpiData analysis V2.2.2.182 for analysis. We employed a trained radiographer cum data entry operators for this purpose. Data entry was also done daily after every camp as he was part of the screening team which accompanied the van. Data entry was regularly checked by the team leader, the nurse.

Proportions were used to summarize the aggregate data and the socio-demographic and clinical characteristics of those who were screened. A trend line was used to compare the case notification rate before and after the intervention in the study district.

### Ethics approval

Ethical approval was obtained from the Institute Review Board of The Medanta - The Medicity Hospital, Gurugram, Haryana. Administrative approval to conduct the study was obtained from the State RNTCP, Haryana.

## Results

The socio-demographic characteristics of the patients screened under strategy 1&2 are given in Table 1.

**Table 1:** Characteristics of patients screened using strategy 1 and 2 in a mobile van intervention in District Mewat, Haryana, India, 2016

Characteristics	Strategy 1	Strategy 2
	*N* (%)	*N* (%)
Total	121 (100)	596 (100)
Sex		
Male	73 (60)	355 (60)
Female	45 (37)	241 (40)
Missing	03 (3)	0 (0)
Age group		
< 15 years	9 (7)	53 (9)
15–44	52 (43)	214 (36)
45–64	52 (43)	246 (41)
65 years and above	8 (7)	83 (14)
Symptoms		
Cough	116 (96)	590 (99)
Hemoptysis	9 (7)	6 (1)
Fever	57 (47)	229 (38)
Weight loss	43 (35)	13 (2)
Abnormal CXR s/o TB	39 (32)	214 (36)

*CXR* = chest x-ray, *TB* = Tuberculosis, *may have multiple responses

### Effects of both the strategies

#### Strategy 1

**Fig. 1:**
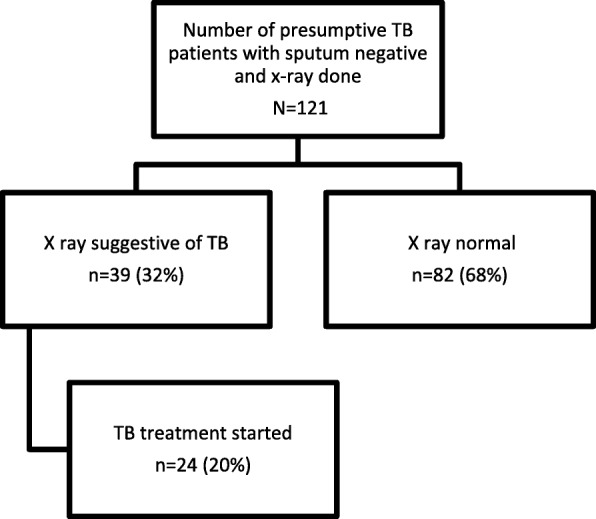
Flow of patients screened by the mobile van strategy 1 in District Mewat, Haryana, India, 2016

A total of 121 smear negative cases were screened with x-ray in nine camps covering four health facilities (3 CHCs and 1 PHC). On an average, 13 patients attended each camp. Nearly 62% of them were males and the mean age of the participants was 45 years. The chest x-rays were suggestive of TB in 39 out of 121 (32%). Of the 39 x-rays suggestive of TB, 25 (64%) had upper lobe infiltrates, 13 (33%) had consolidation and 1 (3%) had cavity. Of these 24 were started on treatment (Fig. 1)**.**

#### Strategy 2

In this strategy, 12 visits were conducted over a 3-week period covering 3 CHCs and 9 PHCs. A total of 596 presumptive TB cases (more than 2 weeks of cough) were screened with chest x-ray. Nearly 60% of them were males and the mean age of the participants was 47 years. The chest x-rays were abnormal and suggestive of TB (old or active) in 208 out of 596 (35%). Of 208, 23 patients were already diagnosed and on TB treatment, and were excluded from further analysis. Of the remaining 185 x-rays, 77 x-rays showing fibrosis and calcification, pleural effusion and lower lobe consolidation were excluded.

**Fig. 2:**
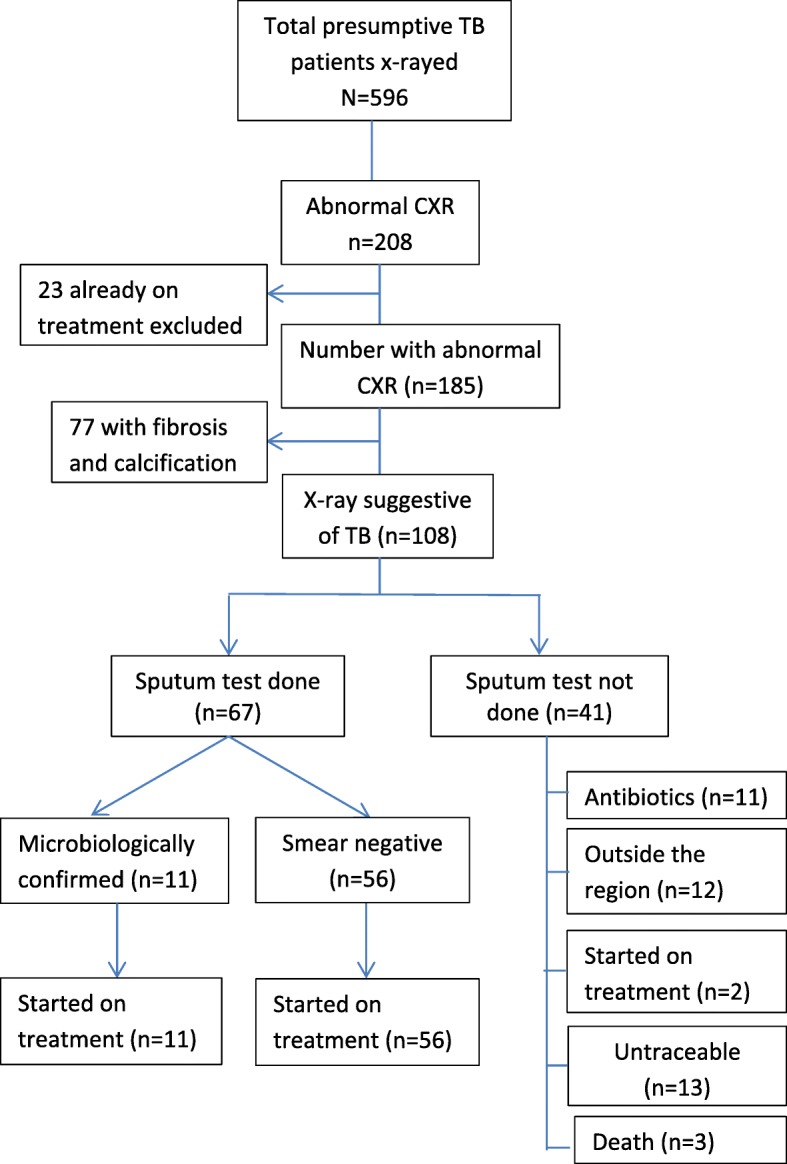
Flow of patients screened by the mobile van strategy 2 in District Mewat, Haryana, India, 2016

Of 108 with suggestive x-ray findings, only 67 underwent sputum testing, of which 11 were smear positive and 56 were smear negative. All 67 were started on treatment. Efforts were made at a later date to locate the remaining 41 patients. Of the ones who could be traced – 2 were started on treatment for TB later, 11 received antibiotics, 12 were out of the region and 2 had died; 13 could not be traced Fig. 2.

### Impact on case notification rate

**Fig. 3:**
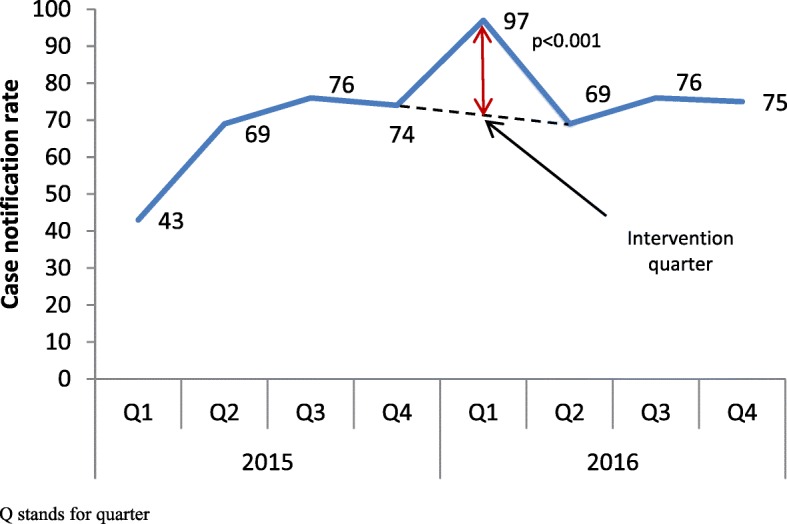
TB Case notification rate of smear negative cases before, during and after the intervention in district Mewat, Haryana, India, 2015–16

**Fig. 4:**
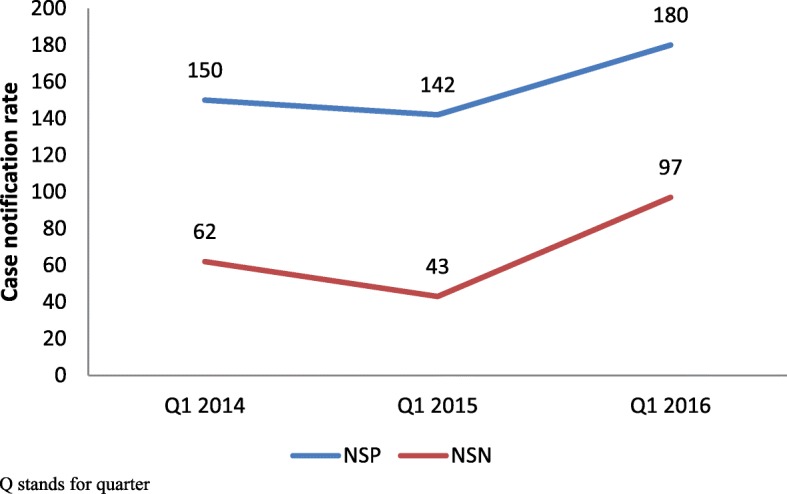
Comparison of TB Case notification rates of both smear positive and negative cases separately in the first quarter of the last two years prior to the intervention in district Mewat, Haryana, India, 2014–16

Figure 3 shows that the case notification rate for new smear negative TB cases was significantly higher (*p* < 0.001) during the intervention period (first quarter of 2016) compared to previous and subsequent quarters. Figure 4 also shows the rise in case notification rates for both new smear positive and negative TB cases during the first quarter of 2016 (intervention period) compared to the first quarters of previous two years.

### Cost-effectiveness analysis

Strategy 1: Cost of screening an individual for TB symptoms was US$ 6 whereas that of identifying a case of TB and initiating on treatment under strategy 1 was US$ 32.

Strategy 2: Cost of screening an individual for TB symptoms was US$ 2 whereas that of identifying a case of TB and starting on treatment was US$ 15 under strategy 2 Table 2.

**Table 2:** Comparison of cost analysis of strategy 1 and 2 per screening, x-ray positivity and per TB case started on DOTS

Service indicators	Strategy 1			Strategy 2		
	13/camp	20/camp	30/camp	50/camp	75/camp	100/camp
Total patients screened annually	2808	4320	6480	10,800	16,200	21,600
Cost per individual screened	6	4	3	2	1	1
Number of x-ray positive	899	1382	2074	2160	3240	4320
Cost per x-ray positive	20	13	9	9	6	5
Number started on DOTS	562	864	1296	1296	1944	2592
Cost per TB case on DOTS	32	21	14	15	10	8

*DOTS* = Directly Observed Treatment Short Course, cost figures expressed in US dollars

### Sensitivity analysis

As part of the sensitivity analysis, we varied the number of persons attending the camp in both the strategies only to find that strategy 2 was cost effective in all scenarios. The cost per screening was US$ 3 and cost for initiating a TB case on treatment was US$ 14 in strategy 1 if 30 persons attend each camp. However, under strategy 2, the cost for screening each individual for TB symptoms was US$ 1, whereas that of initiating a TB case on treatment was US$ 8 if 100 persons attend each camp Table 2.

## Discussion

The study findings highlight the feasibility of a public-private partnership in the provision of digital chest x-ray at places where the facility is not available to identify the missing TB cases in the community. In this model, a mobile van equipped with digital x-ray was provided by a corporate hospital with scheduled visits to peripheral health institutes within a not-for-profit partnership. It worked in close liaison with local healthcare providers and followed the RNTCP diagnostic and treatment algorithm.

Diagnosis of smear negative TB often involves a longer health service delay because of the algorithm which needs 11–34 days to establish a diagnosis under the most optimistic scenarios [10, 11]. Poor access to chest radiography and trained staff at peripheral health facilities and eventual loss-to-follow-up when patients are referred to the higher level for x-ray are some of the reasons for under-diagnosis of smear negative cases. These delays result in individual morbidity as well as continued transmission in the community. The sputum negative patient with Pulmonary TB can infect 4 patients per year [12]. Thus, with the recent revised operational guidelines recommending x-ray as a screening tool alongside sputum examination in the diagnostic algorithm, MXU model looks feasible and scalable at least for the high-risk groups, if not all. This model provides digital x-ray in remote locations along with the trained staff (radiographer and chest physician at the district level) without having the patient to visit higher levels of facility for x-ray.

A previous study by Story et al. concluded that cases of active pulmonary disease identified by the MXU were less likely to be sputum smear positive on diagnosis than passively identified cases from the same populations [13]. This is probably because the MXU identifies people earlier in the course of disease, before they become infectious, thereby limiting the transmission of the disease in the community. Another explanation could be the access to x-ray facilities which renders diagnosis of smear negative disease which otherwise would have been missed. The present study showed a significant increase in the Case Notification Rate (CNR) in smear negative TB in the study district during the period of intervention. Strategy 1 involved diagnosis of smear negative TB by providing x-ray facility to smear negative presumptive TB cases. In strategy 2, among 67 patients who had both x-ray and sputum examination done, 11 were smear positive whereas 56 were diagnosed with smear negative TB which substantiates the above stated fact.

The cost per TB case diagnosed and started on treatment was much higher in other studies evaluating a mobile screening unit compared to our screening strategy, although the population and the algorithm of screening in those previous studies vary [14]. One of the reasons for this difference in cost might be due to the presence of culture in the diagnostic algorithm in addition to sputum smear and x-ray examination.

One of the key findings of the study is the cost difference in screening and identifying a case of TB between both the strategies. One possible reason for this difference could be the fact that many people attended the screening camps under strategy 2 compared to strategy 1 which is probably due to the additional IEC component under strategy 2. A structured IEC campaign involving multiple relevant stakeholders was planned prior to each camp in and around the local area. This mobilized the entire community leading to higher attendance at the camps. Thus, IEC should be an integral component of any similar screening camps in order to increase its yield and cost-effectiveness.

Mobile x-ray unit (MXU) has been shown to be an effective tool for active case finding especially in difficult-to-reach populations which are known to have a high burden of disease. This is because of its ability to identify cases at an early stage of the disease when they are less infectious, thereby cutting the spread of the disease. This strategy of improving access to x-ray using a mobile unit has also been demonstrated to be cost-effective in such populations in previous studies [13, 15].

The study strengths are that it was conducted within the routine health system engaging a PPM model and thus operationally relevant and can be easily applied to other settings, thus allowing scale-up elsewhere. This operational research also responds to an identified national research priority.

There were few limitations in this study. First, in strategy 2, nearly one-third of the patients with x-ray suggestive of TB did not undergo sputum examination. This project is now being scaled-up to all the districts in Haryana with more rigor. Second, there was no information on impact of the intervention on the delay in diagnosis and treatment and their treatment outcomes compared to those detected by passive case finding. Third, cost analysis was done in terms of cost per screening a presumptive TB case or TB case diagnosed which rules out comparison with interventions in other subjects or sectors. Fourth, overdiagnosis of TB, especially smear negative ones as seen in this intervention might overestimate the cost effectiveness of this model.

## Conclusions

The study reports a new initiative within a PPM model to improve the diagnosis of PTB by filling the gap in the current diagnostic infrastructure. The results of our study are encouraging and point towards chest x-ray screening as a viable tool in TB case finding algorithm strategy, both operationally and also in economic terms. Although we believe there is potential for replication of strategy 2 model in other states, we still need more substantial evidence to support our claims. Strategy 2 has now been scaled up in other states as part of the same project and assessment of the same will provide us with the necessary evidence. TB case detection could be improved by adding Xpert machines to the mobile van. However, this needs to be piloted in future studies and scaled-up.

## Additional file

Additional file 1: Comparison of costs of case finding strategy 1 and 2. (DOCX 14 kb)

## Abbreviations

CHC: Community health centre
CNR: Chest notification rate
CXR: Chest radiograph
PHC: Primary health centre
RNTCP: Revised national tuberculosis control programme
TB: Tuberculosis
