# Supplementary material for: Comparison of clinical and cost-effectiveness of two strategies using mobile digital x-ray to detect pulmonary tuberculosis in rural India
Source: BMC Public Health. 2019 Jan 22;19:99. doi: 10.1186/s12889-019-6421-1 (PMC6341675; doi:10.1186/s12889-019-6421-1)
Supplement: Supplementary file 1 — Comparison of costs of case finding strategy 1 and 2. (DOCX 14 kb) [file 12889_2019_6421_MOESM1_ESM.docx]

**Additional file 1: Appendix 1**

**Comparison of costs of case finding strategy 1 and 2**

| **Cost heading** | | **Strategy 1** | **Strategy 2** |
| --- | --- | --- | --- |
| **Van** | Cost of van | 2344 | 2344 |
| **Operating costs** | Van insurance | 391 | 391 |
|  | Maintenance cost | 2344 | 2344 |
|  | Fuel | 938 | 938 |
| **Equipment** | Fabrication | 1786 | 1786 |
|  | Digital reader | 1116 | 1116 |
|  | x-ray machine | 268 | 268 |
| **Personnel** | Radiographer | 2813 | 2813 |
|  | Nurse/coordinator | 2813 | 2813 |
|  | Driver | 2813 | 2813 |
| **IEC** | IEC cost | 0 | 1688 |
| **Miscellaneous** | Miscellaneous cost | 313 | 313 |
| **Total cost** |  | 17935 | 19623 |

Costs are expressed in US dollars; IEC=Information Education and Communication
